# Orexin A-Mediated Modulation of Reproductive Activities in Testis of Normal and Cryptorchid Dogs: Possible Model for Studying Relationships Between Energy Metabolism and Reproductive Control

**DOI:** 10.3389/fendo.2019.00816

**Published:** 2019-11-22

**Authors:** Loredana Assisi, Alessandra Pelagalli, Caterina Squillacioti, Giovanna Liguori, Chiara Annunziata, Nicola Mirabella

**Affiliations:** ^1^Department of Biological Sciences, University of Naples Federico II, Naples, Italy; ^2^Department of Advanced Biomedical Sciences, University of Naples Federico II, Naples, Italy; ^3^Institute of Biostructures and Bioimages, National Research Council, Naples, Italy; ^4^Department of Veterinary Medicine and Animal Productions, University of Naples Federico II, Naples, Italy; ^5^Department of Pharmacy, University of Naples Federico II, Naples, Italy

**Keywords:** orexin A, reproduction, dog, energy, metabolism, testosterone

## Abstract

Orexin A (OxA) is a neuropeptide produced in the lateral hypothalamus that performs pleiotropic functions in different tissues, including involvement in energy homeostasis and reproductive neuroendocrine functions. The role of OxA is particularly important given the well-studied relationships between physiological mechanisms controlling energy balance and reproduction. The enzyme P450 aromatase (ARO) helps convert androgens to estrogens and has roles in steroidogenesis, spermatogenesis, and energy metabolism in several organs. The goal of this study was thus to investigate the role of OxA in ARO activity and the effects of this regulation on reproductive homeostasis in male gonads from healthy and cryptorchid dogs. The cryptorchidism is a specific condition characterized by altered reproductive and metabolic activities, the latter of which emerge from impaired glycolysis. OxA helps to stimulate testosterone (T) synthesis in the dog testis. We aimed to investigate OxA-mediated modulation of 17β-estradiol (17β-E) synthesis, ARO expression and metabolic indicators in testis of normal and cryptorchid dogs. Our results indicate putative effects of OxA on estrogen biosynthesis and ARO activity based on western blotting analysis and immunohistochemistry for ARO detection and *in vitro* tests. OxA triggered decrease in estrogen production and ARO activity inhibition; reduced ARO activity thus prevented the conversion of T to estrogens and increasing OxA-mediated synthesis of T. Furthermore, we characterized some metabolic and oxidative modulations in normal and cryptorchid dog's testis. The steroidogenic regulation by OxA and its modulation of ARO activity led us to hypothesize that OxA is a potential therapeutic target in pathological conditions associated with steroidogenic alterations and OxA possible involvement in metabolic processes in the male gonad.

## Introduction

Spermatogenesis is a biological process in animals that requires additional energy stores for performance. An important prerequisite for the success of spermatogenesis is that Sertoli and germ cells cooperate for metabolic pathway activation to ensure adequate lactate concentrations inside testis tubule lumen (1), as confirmed by studying the etio-pathogenesis of different diseases. For example, diabetes characterized by a hyperglycemic condition is associated with male infertility (2). Numerous additional studies have demonstrated direct interactions between spermatogenesis anomalies, infertility disorders, and high estradiol (E) levels. In addition, androgens, estrogens, and other testicular factors appear to have important roles in metabolic process control of testis. Among these factors, 17β-estradiol (17β-E) plays a key role in testis function-related mechanisms (3) including spermatogonia division, spermatid differentiation, acrosome biogenesis, sperm motility (4, 5), and Sertoli cells (SCs) metabolism through modulation of glucose metabolism (6). Moreover, estrogens are involved in negative feedback of the pituitary gland to control gonadotropin secretion, hence a lack of estrogen and inappropriate estrogen exposure disturbs the delicate metabolic balance of the hypothalamic—pituitary—testis axis (4). The P450 enzyme aromatase (ARO) plays a pivotal role in these processes by irreversibly aromatizing androgens into estrogens that directly interact with cell surface receptors (4, 7–9). ARO is an enzymatic complex composed of a ubiquitous NADPH-cytochrome P450 reductase and a cytochrome P450 aromatase, which contains the steroid-binding site (9). This enzyme complex is localized to the endoplasmic reticulum of many different areas of the body, including the testis (10), in which its distribution changes during development, being primarily located within Sertoli cells in immature animals and in Leydig and germ cells of mature animals (7, 9, 11, 12). The presence of ARO in the testes has been reported for numerous animal species (9, 12–18). ARO also regulates glucose metabolism (19), which is critical for spermatogenesis. Thus, in light of its importance, ARO activity must be finely regulated (3, 20).

Orexin A (OxA) also appears to play a role in spermatogenesis control and glucose homeostasis by modulating glucose transporter 3 (Glut3), as demonstrated using *in vitro* and *ex vivo* studies of neonatal mice (21, 22). OxA is a hypothalamic neuropeptide and specifically regulates portions of the reproductive axis. The peptides OxA and orexin B (OxB) are derived from the proteolytic cleavage of a prepro-orexin precursor and bind the receptors orexin receptor 1 (OX1R) and orexin receptor 2 (OX2R). Prepro-orexin, OxA, and OX1R have been identified in rat testis and epididymis (23–27), alpacas (*Vicugna pacos*) (28, 29), mouse testis (21, 30), the normal and cryptorchid male gonads (31), and the urethro-prostatic complex of cattle (32), and in the normal, hyperplastic, and neoplastic prostate of human males (33–35). The effects of OxA on testis function and glucose homeostasis may find useful extrapolations by considering male infertility conditions like cryptorchidism (36). Cryptorchidism is characterized by the failure of one (unilateral) or both (bilateral) testis to descended into the scrotum (37–39) and occurs at particularly high frequencies in dogs, stallion and boars (38, 40). This disorder is accompanied by serious structural and functional alterations of tubular and interstitial components of testis (41, 42) as well as clear alteration of Sertoli cell metabolism accompanied by intracellular lipid accumulation (43). Much recent research has focused on regulatory processes governing the hormonal and metabolic basis of cryptorchidism and their consequences for testis tumor development (44–46). Moreover, cryptorchidism could be considered a representative *in vivo* model of spermatogenesis failure for analyzing complex regulatory mechanisms and testing drug-driven regenerative effects of spermatogenesis (47).

We recently demonstrated a possible relationship between OxA and ARO expression in alpaca testis (48), thus it is evident that OxA, is able to significantly decrease basal 17β-E secretion and OxA acts through decreasing ARO activity. This motivated us to better investigate the possible interaction of OxA and ARO in a disease such as cryptorchidism. In this study we explored ARO expression in normal and cryptorchid canine testis and the effects of OxA stimulation on ARO and 17β-E biosynthesis. Moreover, we characterized metabolic modifications in order to investigate the possible relationship between energy metabolism and reproductive control in cryptorchid condition.

## Materials and Methods

### Antibodies and Chemicals

Rabbit polyclonal anti-cytochrome P450 (aromatase) antibody (ABIN3023082) was purchased from antibodies-online.com (Aachen, Germany). Rabbit anti-glucose transporters (Glut) 3 antibody (sc-74399) was from Santa Cruz Biotechnology (Santa Cruz, CA, United States); anti-rabbit phopsho AKT (Ser473) (#4060), anti-mouse AKT (#2920) and anti-rabbit superoxide dismutase (SOD)2 (D3X8F) (#13141) antibodies were from Cell Signaling Technology (Danvers, MA, United States), biotinylated goat anti-rabbit (BA-1000) secondary antibody, peroxidase-conjugated rabbit anti-goat (PI-9500) IgG, VECTASTAIN ABC kit (PK-6105), and 3,3′-diaminobenzidine tetra-hydrochloride (DAB) solution were obtained from Vector Laboratories (Burlingame, CA, USA); peroxidase-conjugated goat anti-rabbit IgG (111-035-003) and peroxidase-conjugated goat anti-mouse IgG (115-035-003) were purchased form Jackson ImmunoResearch Laboratories Inc. (West Grove, PA, United States).

The peptide OxA (003-30) was obtained from Phoenix Pharmaceuticals Inc. (Karlsruhe, Germany) and the OX1R antagonist SB-408124 was obtained from Sigma Aldrich (Saint Louis, MO, United States). Luteinizing hormone (LH) from sheep pituitary (L5269), monoclonal anti-actin antibody (A4700) and bovine serum albumin (BSA) were purchased from Sigma Chemical Co. (St. Louis, MO, United States). Qproteome formalin-fixed paraffin-embedded (FFPE) tissue kits were purchased from Qiagen (Hilden, Germany). DC protein assay kit was purchased from Bio-Rad Laboratories (Hercules, CA, United States). The enhanced chemiluminescence kit (RPN 2109) was bought from ECL Amersham (Little Chalfont, Buckinghamshire, UK), the marker proteins obtained from Prosieve quadcolor (London, United Kingdom), and the estradiol ELISA kit (DKO003) purchased from Diametra (Perugia, Italy).

### Animals and Tissue Collection

A total of 10 sexually mature dogs with normal testes (*n* = 5) and unilateral cryptorchid testes (*n* = 5) were enrolled in the study. Contralateral descended testes were recovered from each animal. For our experiments tissue samples were divided in three groups: normal testis (testis from normal dogs) (NT), contralateral testis (scrotal testis from dogs affected by unilateral cryptorchidism) (CLT), and cryptic testis (retained testis from dogs affected by unilateral cryptorchidism) (CT). All dogs were mixed-breed, medium-sized, and aged 2–8 years. Obese dogs were excluded from the study. Dogs affected by unilateral cryptorchidism were obtained from the surgery unit of the Department of Veterinary Medicine and Animal Productions of the University of Naples Federico II. Animal care was maintained during surgical procedures and the experimental research protocols were approved by the Ethical Animal Care and Use Committee of the University of Naples Federico II, Department of Veterinary Medicine and Animal Production, Naples, Italy (no. 0005275). Testes were collected immediately after bilateral orchiectomy via surgery. For immunodetection studies, fresh segments of testis were immediately fixed in Bouin's solution containing formaldehyde, picric acid (saturated), and glacial acetic acid, which is preferred for small biopsies (49). For *in vitro* and aromatase activity tests, fresh segments of testis were immediately used or frozen on dry ice and stored at −80°C.

### Immunohistochemistry

After fixation in Bouin's solution, samples were dehydrated in a series of ascending alcohol concentrations, embedded in Paraffin, and cut into 3–6 μm sections for immunohistochemistry and into 15-μm sections for Western blotting. After deparaffinization and hydration, sections were immersed in citric buffer (pH 6.0) for antigen retrieval (50). Tissue sections were then stained using the ABC method as described elsewhere (51, 52). Rabbit polyclonal anti-ARO (1:200) and anti-Glut3 (1:250) antibodies were used as the primary antibody. Sections were incubated with DAB solution until desired intensity of staining was reached then counterstained with hematoxylin for improved identification of cytotypes. Finally, sections were dehydrated with ascending alcohols and mounted with Eukitt. Slides were observed using a Leica DMRA2 microscope and negative controls obtained by omitting the primary antiserum used. To ensure that the immunohistochemical data from control, cryptorchid, and contralateral testis could be compared, sections were processed under same conditions. In addition, immunohistochemistry reactions were performed in triplicate to confirm results.

### Protein Extraction and Western Blotting Analysis

For western blot analysis of ARO, proteins were extracted from tissue sections that had been paraffin-embedded using a Qproteome FFPE tissue kit, which creates optimized conditions for intact total protein extraction from FFPE tissues (53). Briefly, 12 tissue sections (each 15-μm thick) were deparaffinized in xylene, rehydrated in a graded alcohol series and mixed with 100 μl of extraction buffer supplemented with β-mercaptoethanol and 1x Protease and Phosphatase Inhibitors Cocktail (Sigma). To evaluate the expression of Glut3, SOD2, phospho-AKT and total AKT, frozen testis from normal and cryptorchid dogs were used to obtain other protein samples. After homogenization, testis were lysed in lysis buffer according to the method previously described (54). Then, the protein samples were cooled on ice for 5 min, with continuous shaking, and incubated at 100°C for 20 min followed by incubation at 80°C for 2 h on a heating block. The samples were centrifuged at 14,000 × g at 4°C for 15 min to collect total proteins, whose concentrations were determined with the Bradford protein assay performed according to the manufacturer's protocol.

Western blotting was performed as described elsewhere (55). Briefly, the same total amount of protein per lane was loaded for each sample then separated using 10% SDS-PAGE gels and NuPage Bis-Tris 4–12% gradient gels (Invitrogen, Carlsbad, CA, United States) under reducing conditions. Proteins were transferred onto nitrocellulose membranes using the iBlot system from Invitrogen (Carlsbad, CA, United States). Blots were then probed with rabbit polyclonal anti-ARO antibody (1:4,000), anti-Glut3 antibody, anti-SOD2, anti- phopsho AKT and AKT (all diluted 1:1,000) and then with secondary anti-rabbit and anti-mouse IgG antibodies (1:2,000). Western blot for β-actin was performed to ensure equal sample loading. Protein detection was performed with the ECL Plus Western blotting detection system according to the manufacturer's instructions. Band intensities were quantified using ImageJ software (NIH, Bethesda, United States) as needed.

### *In vitro* Determination of 17β-Estradiol Levels

Fresh testis samples of each type were de-capsulated, cut into small pieces (250 mg/piece) and distributed into sample tubes (one piece per well). After the addition of 2 ml KRB buffer (10 mM glucose, 100 μM bacitracin, 0.1% ascorbic acid, 0.1% BSA), tubes were incubated for 60 min at 37°C, 95% oxygen, and 5% CO_2_ with shaking at 60 cycles/min. Media was then replaced with 2 ml fresh KRB buffer and 1 nM of test substance. The test substance for the first group was the KRB buffer-only control, for the second group was OxA, and for the last group the OX1R antagonist, SB-408124 (specific for OxA), and OxA were added. All sample tubes were incubated at 37°C for 12 h. After incubation, ethyl ether was added to each sample and tubes shaken vigorously. The samples were left 4°C for 10 min and supernatant collected. Supernatant-containing tubes were dried overnight at room temperature then the residue in each tube dissolved in 0.5 ml 0.05 M PBS, pH 7.5, containing 10 mg/ml BSA. The 17β-estradiol levels were determined using the 17β- estradiol ELISA kit according to the manufacturer's instructions. The following limits of detection were used: sensitivity 4 pg, intra-assay variability 4.9%, inter-assay variability 6.8%. The rate of E recovery from testis was about 85%.

### Aromatase Activity Assay

ARO activity was evaluated by measuring the *in vitro* conversion rate of testosterone to 17β-estradiol using fresh tissue. One piece of testis sample was placed in each well of a multi-well plate and the above reported substances was added. Testosterone at a concentration of 35 μM dissolved in 100 μl of 3 mg/ml NADPH solution was then added to each well. Suspensions were then incubated in a shaking bath as previously described (48) and then rapidly frozen. Well contents were then extracted three times using ether. Solvents were pooled and air-dried and 17β-E levels determined in extracted residues using ELISA, as previously reported (48). Results are expressed as the 17β-E concentration produced per g of tissue and per hour.

### Statistical Analysis

Data from *in vitro* tests were compared by two-way ANOVA followed by Duncan's test for multi-group comparison. All data were expressed as mean ± S.D. (standard deviation) of at least three different experiments and each experiment performed either in duplicate or triplicate. The level of significance was set at *p* < 0.01.

## Results

**Figure 1:**
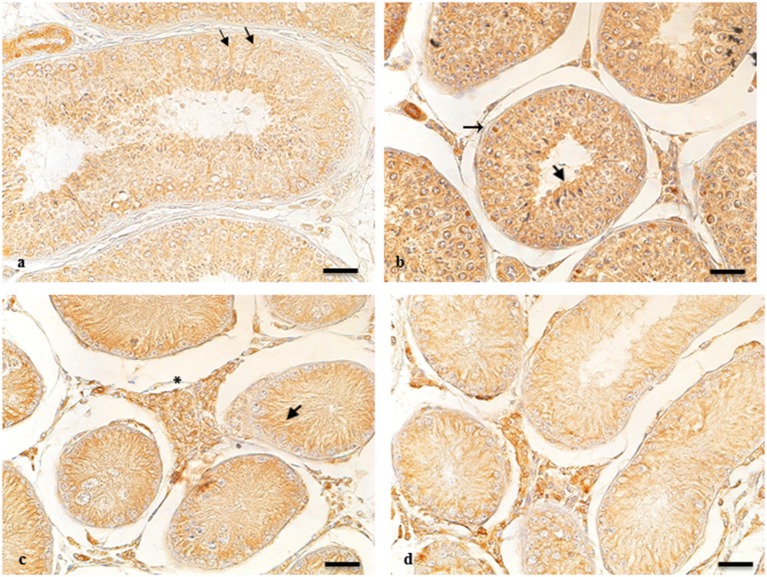
ARO-IR in cytotypes of normal and cryptic gonad of dogs. **(a,d)** Sertoli cells containing positive granules describing entire cellular profiles in normal and cryptic canine testis (arrow and arrowhead, respectively). **(b)** ARO-IR occurs as a single, intensely stained, granular structure, and is contained in the perinuclear cytoplasm of some spermatogonia (arrow). **(c)** a cluster of Leydig cells containing different quantities of reactive material in their cytoplasm in the retained male gonad (asterisk). Bar: 25 μm.

ARO-immunohistochemistry results are described in Figure 1. The reactive material showed a granular aspect and cytoplasmic localization in the Leydig cells from normal and cryptic gonads (Figures 1a,c). These cell types were numerous and often organized in small groups composed of cells with different degrees of staining.

For NT, ARO-immunoreactivity (IR) was observed in the basement membrane of the seminiferous tubule of spermatogonia (Figure 1b). Seminiferous tubules of the CT were composed of mostly Sertoli cells, in which perinuclear expression of ARO was observed. ARO-IR in the CLT was similar to that in NT (data not shown).

**Figure 2:**
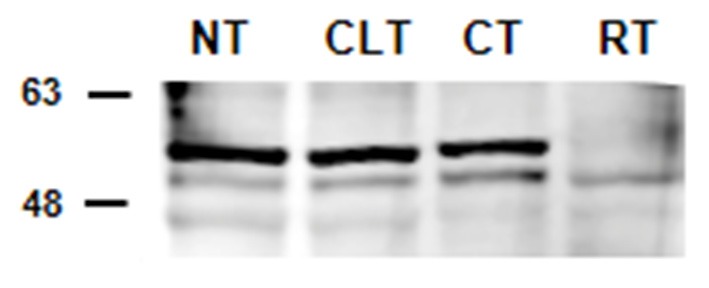
Detection by western blot analysis of ARO in the normal, contralateral, and cryptic male gonads of dogs. ARO was detected in the normal (NT), contralateral (CLT), and cryptic (CT) testis. Samples from rat testis (RT) were also examined as positive controls. In all the examined tissues, ARO appears as 2 bands, one at ~49 kDa and another ~51–53 kDa. This image is representative of three different experiments.

The results of western blot analysis are shown in Figure 2. Testicular extracts from dogs showed reactions with the anti-cytochrome P450 (ARO) antibody, which yielded a strong band of 51–53 kDa and a weaker band at 49 kDa (Figure 2). Similar banding profiles were observed in protein extracts from rat testis, although the band at 51–53 kDa from these samples showed relatively weaker signals (49 kDa).

**Figure 3:**
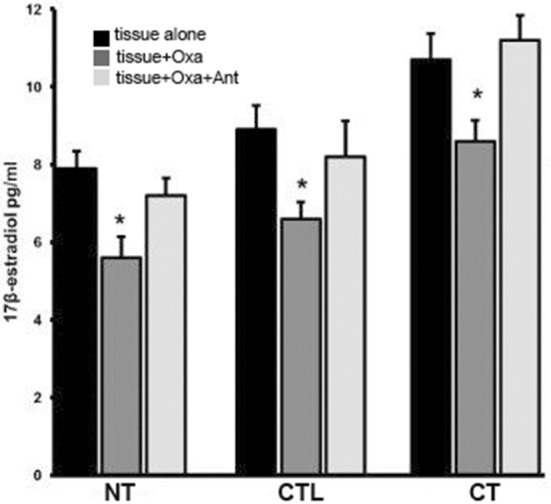
17βE secretion *in vitro* carried out in normal, cryptorchid, and contralateral dog testis. Testicular slices were incubated with OxA alone or with OxA and the OX1R antagonist SB-408124 and the 17βE level in the media monitored after 12 h. Values are normalized per ml of incubation medium. Data are expressed as mean ± SD (*n* = 5 samples/group), *p < 0.01.

Figure 3 shows results from *in vitro* experiments in which normal, controlateral, cryptorchid dog testis slices were incubated with medium containing OxA alone or both OxA and the OX1R antagonist SB-408124. Tissue slices incubated with medium only were used as controls of this experiment. High levels of 17-βE were observed in CT slices relative to the basal levels of NT treated with medium only. OxA significantly decreased basal levels of 17-βE secretion in all three tissue types. In NT, after 12 h of OxA treatment, 17-βE levels were lower than those of control testis (from 7.9 ± 045 to 5.6 ± 0.54 pg/g tissue, *p* < 0.01 tissue vs. control). Conversely, the antagonist SB-408124 nullified the OxA-induced drop in 17-βE levels (from 5.6 ± 0.54 to 7.2 ± 045 pg/g tissue, *p* < 0.01 vs. with OxA alone). This trend also occurred for the other two testis types. In CT, OxA reduces the 17βE level from 10.7 ± 0.67 to 8.6 ± 0.5 pg/g tissue and OxA antagonist presence increases the final levels of 17-βE levels to 11.2 ± 0.64 pg/g tissue. Finally, in the CLT OxA induces a decrease in 17βE levels from 8.9 ± 0.62 to 6.6 ± 0.43 pg/g tissue and OxA antagonist presence decreases the post-treatment 17-βE level to 8.2 ± 0.92 pg/g tissue.

**Figure 4:**
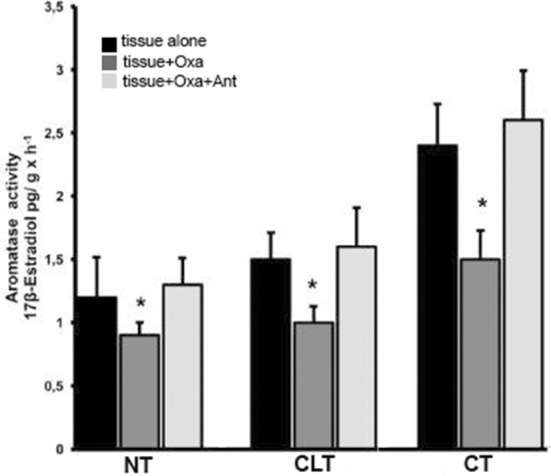
ARO activity evaluated with *in vitro* tests carried out in normal, cryptorchid, and contralateral dog testis. Testicular slices were incubated in presence of testosterone alone, with OxA, or with both OxA and the OX1R antagonist SB-408124 and 17βE production monitored. Values are normalized per g tissue per hour. Data are expressed as mean ± SD (*n* = 5 samples/group), *p < 0.01.

Figure 4 shows the ARO activity of all three tissue types in the presence of substrate (T) and either OxA alone or OxA and the OX1R antagonist. ARO activity was evaluated based on 17β-E production via exogenous T conversion. The basal levels of ARO activity are higher in CT than in NT (2.4 ± 0.33 compared to 1.2 ± 032 pg 17βE per g tissue per hour, *p* < 0.01 vs. control). In presence of OxA the ARO activity of NT decreases while the presence of the antagonist SB-408124 prevents this decrease. More specifically, OxA induces a decrease in ARO activity, from 1.2 ± 0.32 to 0.9 ± 0.1 pg of 17βE per g tissue per hour (*p* < 0.01 normal testis alone vs. normal testis with OxA), while presence of the antagonist SB-408124, ameliorates this effect (from 0.9 ± 0.1 to 1.3 ± 0.21 pg of 17βE per g tissue per hour, *p* < 0.01 normal testis with OxA vs. normal testis with both OxA and antagonist). This trend is also evident also for CT and CLT. In CT, OxA decreases the level of 17-βE from 2.4 ± 0.33 to 1.5 ± 0.23 pg of 17βE per g tissue per hour (*p* < 0.01 cryptorchid tissue alone vs. cryptorchid tissue with OxA) while the antagonist SB-408124 reduces this decrease (from 1.5 ± 0.23 to 2.6 ± 0.39 pg of 17βE per g tissue per hour, *p* < 0.01 cryptorchid tissue with OxA vs. cryptorchid tissue with OxA and antagonist). The same effect is observed for CLT.

**Figure 5:**
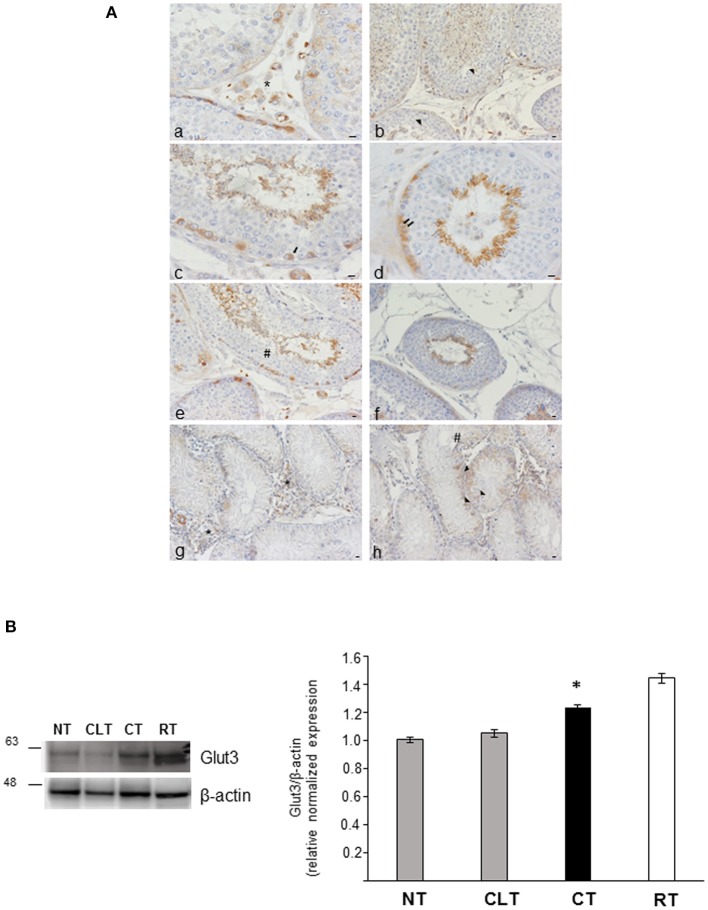
**(A)** Glut3-IR in cytotypes of normal and cryptic gonad of dogs. (a,g) A large group of Leydig cells positive to Glut3 in NT and CT (asterisk) were found; (b,h) Glut3-IR were described in the apical and basal portions of the cytoplasm of Sertoli cells (arrowhead) in NT and CT; (c,d) few spermatogonia (c) and resting or pre-leptotene spermatocytes (d) positive to Glut3 were found along the basal membrane of the testicular tubule of NT (arrow and double arrow, respectively); (e,f) round or immature (e) and elongated or mature (f) spermatids were localized toward the tubular lumen (hashtag) in TN. Bar: 50 μm. **(B)** Western blot analysis of Glut3 protein expression level in the canine NT, CLT, and CT. Samples from rat testis (RT) were blotted as positive controls. In all the examined tissues, Glut3 appears as a band at ~ 56 kDa. A significative increase in Glut3 expression is observed in CT respect to NT and CLT (densitometric analysis). Data are expressed as mean ± SD (*n* = 5 samples/group), *p < 0.01.

To investigate the relationship between energy metabolism and reproductive function in canine cryptorchidism, we evaluated Glut3 localization in testis and its expression by western blotting. As shown in Figure 5A, Glut3-IR was found in the tubular and interstitial compartment both in NT and CT samples. More in detail, Glut3-IR was found in Leydig cells characterized by punctiform distribution of this glucose transporter in the cytoplasm (Figure 5Aa). In the Sertoli cells, Glut3-IR was predominantly localized in the apical and basal portions of the cytoplasm and rarely described the entire profile of this cytotype (Figure 5Ab). Glut3-IR was also evidenced in few spermatogonia localized along the basal membrane (Figure 5Ac), in resting pre-leptotene spermatocytes (Figure 5Ad) and in round or immature (Figure 5Ae) and elongated or mature spermatids (Figure 5Af). In these latter cell types Glut3-IR was observed in the apical portion of the cytoplasm toward the lumen. Interestingly, in CT samples, Glut3-IR was found in a large group of Leydig cells that were intensely stained (Figure 5Ag). In Sertoli cells, Glut3-IR was mostly distributed in the basal portion of the cells and the entire profile was described only in few cells (Figure 5Ah). Glut3-IR in the CLT was similar to that in normal testis (data not shown).

**Figure 6:**
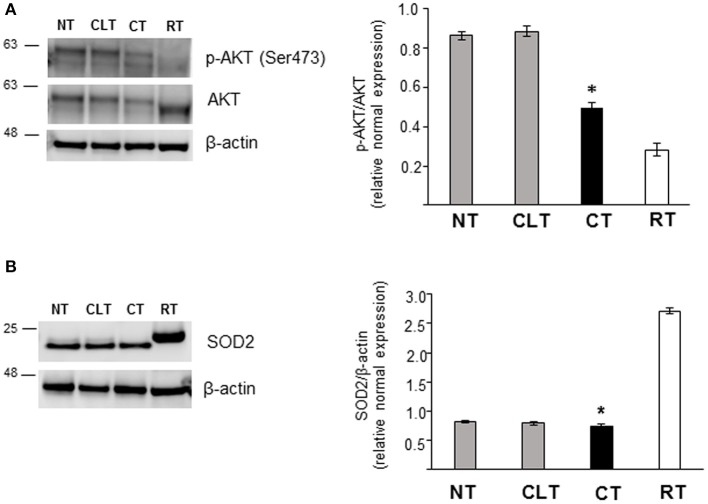
Western blot analysis of phospho-AKT, AKT, and SOD protein expression in normal, cryptorchid, and contralateral dog testis. **(A)** Phospho-AKT/AKT and **(B)** SOD2 proteins are evidenced respectively as a band of 60 and 22 kDa in all the tested tissues. Densitometric analysis shows a significant decrease in both protein expression level in CT compared with NT and CTL. Samples from rat testis (RT) were blotted as positive controls. Data are expressed as mean ± SD (*n* = 5 samples/group), *p < 0.01.

As shown in Figure 5B, CT displayed an increased Glut3 protein expression compared to NT and CLT. To evaluate further modifications related to cryptorchid condition, western blotting analysis of phosphorylation levels of AKT and of the SOD2 protein expression was performed. As shown in Figure 6A, a significant decrease of phosphorylated protein AKT was found in CT respect to NT and CLT. Similarly, SOD2 protein showed a decrease in its expression in CT respect to NT and CLT (Figure 6B).

## Discussion

The goal of this study was to investigate the presence of ARO in the dog testis both under normal and cryptorchid conditions and evaluate the effects of OxA on this enzyme and estrogen (E) biosynthesis. Results from our studies demonstrated potential modulation of ARO activity by OxA in testis of both normal and cryptorchid dogs. We first aimed to demonstrate the presence of ARO in dog testes by immunohistochemistry and showed enzyme localization in both interstitial and tubular compartments of normal and cryptic testis. Western blotting analysis confirmed immunohistochemical results and identified two antibody-specific bands at ~51–53 and 49 kDa in agreement with previous results from Lambard et al. (56). We previously demonstrated the presence and expression of OxA and OxA receptor 1 in testis from normal and cryptorchid dogs (31). OxA was seen in both the interstitial and tubular compartments. The immunohistochemical co-localization of OxA and ARO in most testicular cytotypes may be ascribed to a functional relationship between these proteins in regulating spermatogenesis. Leydig cells immunoreactive to OxA andARO serve as important sites for activating the downstream signaling processes during steroidogenesis. The balance between expression of ARO and sex hormones is pathologically altered in cryptorchidism. Cryptorchidism is a reproductive disease in which one or both testes fail to descend toward the scrotum. This condition characterized by a gradual decrease in the volume of seminiferous tubules per testis and leads to a marked reduction (>50%) of luminal volume of the seminiferous tubule with causing morphological and metabolic alterations (57).

Modifications from cryptorchidism also concern steroidogenesis, as basal 17β-E levels in cryptorchid tissue are higher than those in the normal tissue, leading to infertility (58). Furthermore, E levels decrease in the presence of OxA, demonstrating an inhibitory effect of OxA on E synthesis. A possible role of OxA in these mechanisms was suggested in previous studies demonstrating the presence and expression of OxA in the testis of several species (25, 28, 31), according to which OxA induces T secretion via binding OX1R. OxA is also involved in down-regulating estrogen secretion. The underlying mechanism of these effects is most likely reduced ARO activity, which further hinders the conversion of T to E and consequently increases OxA-triggered T synthesis. These two events may be related since both are eliminated by the presence of the OX1R antagonist SB-408124. On this basis, we can hypothesize a combined role of OxA and ARO in the testicular tubular compartment for regulation of steroidogenesis and spermatogenesis.

To further elucidate this relationship between the increase of T levels and 17β-E, we directly investigated the effects of OxA on ARO activity, which is the principal source of E in mammalian testis. ARO inhibition is associated with lowered E and elevated levels of gonadotropins and T via the E-sensitive male hypothalamus-pituitary-gonadal axis, which potentially stimulates sperm production (59). Our *in vitro* tests showed that the OxA inhibits ARO activity in all three types of tissue examined and that this is mediated by OX1R, since this effect is lost in the presence of the OX1R antagonist SB-408124. These findings confirm those previously described for alpaca male gonads (48), in which OxA-triggered downregulation of E secretion may be ascribed to ARO inhibition by exogenous OxA. This OxA-induced downregulation of E may be caused by reduced ARO activity, potentially due to repressed expression. This aspect implies that OxA may indirectly regulate ARO expression, causing decreased E levels and stimulating T production.

Recently, it has been demonstrated that OxA plays a role at testicular level on glucose metabolism acting on Glut 3 regulation and Glut 8 expression (22). Glut3 was previously described in different cytotypes in human (60, 61), mouse (62), and rat (63) testes and both in normal and cryptic male gonad of the dog (64). In cryptorchid condition, the intensely stained and numerous Leydig cells positive to Glut3 confirmed the previous finding reported by Hann et al. (64). In the present study, Glut3 was described not only in Leydig and SC in NT and CT samples, but also in the tubular compartment.

These morphological findings was confirmed at molecular level. In CT samples, the increased Glut3 protein expression, could suggest a putative role of this glucose transporter in this pathological condition. Our data are in agreement with previous data showing that the increase expression of Glut3 in the Leydig cells may be associated with cell hyperplasia and the relevant metabolic role of this glucose transporter in retained gonad (64). Interestingly, the cryptorchid condition predisposes to testicular germ cell tumors (TGCTs), characterized by metabolic hyper-glycolitic cell phenotype, promoting tumor development (65, 66).

Moreover, cryptorchidism, as well the impairment of male infertility (67), can be associated to elevate oxidative status (68, 69). Here, we showed that SOD2 protein expression in canine cryptorchid condition was significantly reduced compared to NT.

Kawakami et al. (70) have already demonstrated that SOD activity was down regulated in unilateral cryptorchid testis of dog affected by Sertoli cell tumor. Our finding supports the beneficial role of SOD anti-oxidative defense in maintaining sperm fertilization, counteracting the elevated oxidative species revealed in cryptorchid condition (71, 72).

Our data are the first reports to our knowledge showing that canine cryptorchid testis condition is characterized by a reduction of both SOD2 activity and AKT phosphorylation. It has been demonstrated that the decreased phosphorylation level of AKT in CT could be associated to germ cell apoptosis due to the altered oxidative stress balance (73). Since the protective role of OxA during oxidative stress has been recently investigated (74, 75), we may hypothesize a possible involvement of OxA as a key modulator of cryptorchid disease.

These data suggest that OxA could act as a sensor of energy status, participating as a local regulator to maintain a consistent relationship between the T and E. Aromatization is a process that requires energy consumption, therefore OxA downregulation of ARO activity is extremely important to avoid unnecessary glucose consumption.

The possible role of ARO in the regulation of metabolism in breast cancer has recently been investigated by Buch et al. (76). Additionally, ARO has been shown to play roles in glucose and insulin metabolism.

In conclusion, this research demonstrates the relationship of OxA and ARO in male gonads from NT and CT, and its involvement in downregulation of E secretion likely through repressed ARO activity.

On the other hand, Ox-A may be involved in the regulation of metabolic changes occurring in cryptorchid condition, even if further molecular studies are needed to confirm its autocrine and paracrine effects.

All these findings also suggest the translational importance of this canine model that mirrors human pathological.

This consideration derives from studies demonstrating similar profiles of canine and human epididymal proteins at the molecular level measured in terms of tissue distribution, relative abundance, and spatial patterns within tissues (77).

## Data Availability Statement

All datasets generated for this study are included in the article/supplementary material.

## Ethics Statement

Ethics approval was obtained from the Ethical Animal Care and Use Committee of the University of Naples Federico II, Department of Veterinary Medicine and Animal Production, Naples, Italy (no. 0050377). Written informed consent was obtained from the owners for the participation of their animals in this study.

## Author Contributions

All authors conceived of the presented idea. GL, CS, AP, and NM contributed to tissue sample collection and preparation. LA designed and performed the experiments of *in vitro* tests and ARO activity assay. GL, AP, CS, and CA designed and performed the experiments of Western blot and immunohistochemistry. All authors contributed to interpretation and analysis of the data. All authors provided critical feedback and helped shape the research, analysis and manuscript.

### Conflict of Interest

The authors declare that the research was conducted in the absence of any commercial or financial relationships that could be construed as a potential conflict of interest.
